# A Perspective on Client-Psychologist Relationships in Videoconferencing Psychotherapy: Literature Review

**DOI:** 10.2196/19004

**Published:** 2021-02-19

**Authors:** Francesco Cataldo, Shanton Chang, Antonette Mendoza, George Buchanan

**Affiliations:** 1 School of Computing and Information Systems University Of Melbourne Melbourne Australia

**Keywords:** videoconference, psychotherapy, professional-patient relations, client-psychologist relationships, therapeutic alliance, telehealth, mobile phone

## Abstract

**Background:**

During the COVID-19 pandemic, people have been encouraged to maintain social distance. Technology helps people schedule meetings as remote videoconferencing sessions rather than face-to-face interactions. Psychologists are in high demand because of an increase in stress as a result of COVID-19, and videoconferencing provides an opportunity for mental health clinicians to treat current and new referrals. However, shifting treatment from face-to-face to videoconferencing is not simple: both psychologists and clients miss in-person information cues, including body language.

**Objective:**

This review proposes a new theoretical framework to guide the design of future studies examining the impact of a computer as a mediator of psychologist-client relationships and the influence of videoconferencing on the relationship process.

**Methods:**

We conducted a literature review including studies focused on communication and key concepts of the therapeutic relationship and therapeutic alliance.

**Results:**

Studies have reported that clients are generally satisfied with videoconference therapy in terms of the relationship with their therapists and the establishment of the therapeutic alliance. Conversely, studies indicate that psychologists continue to highlight difficulties in establishing the same quality of therapeutic relationship and therapeutic alliance. The contrasting experiences might underlie the differences in the type of emotional and cognitive work required by both actors in any therapy session; furthermore, the computer seems to take part in their interaction not only as a vehicle to transmit messages but also as an active part of the communication. A new model of interaction and relationship is proposed, taking into account the presence of the computer, along with further hypotheses.

**Conclusions:**

It is important to consider the computer as having an active role in the client-psychologist relationship; thus, it is a third party to the communication that either assists or interferes with the interaction between psychologists and clients.

## Introduction

### Background

Since the beginning of 2020, the COVID-19 health pandemic has induced people to avoid face-to-face interactions. The phenomenon is impacting several professions, and features of videoconference (VC) platform have been currently adapted for business meetings. In the case of psychologists, professional association boards across the world have encouraged professionals to keep treating clients using videoconference technologies (VCTs). The assumption is that VCTs might also help clients who live in rural and remote areas, extending access to mental health services [1].

Psychotherapy sessions are traditionally delivered face-to-face in the psychologist’s consultation room. In this physical space, the therapist and the client start to build their relationship and create strong mutual trust. The uninterrupted sharing of a physical space enables the therapist to identify clients’ reactions and vice versa. Moreover, presence enables therapists to be at once *physically, emotionally, cognitively, spiritually, and relationally* [2] in touch with themselves and the clients. Thus, this concrete experience of presence becomes therapeutic and enables clients to experience neurophysiological safety, and consequently, their relationship is enhanced, and the healing process is favored [3].

According to Rogers [4-6], presence is a crucial factor in therapy: it allows psychologists and clients to connect by experiencing the same moment, permits the development of empathy, and leads therapists to develop a *therapeutic relationship* (TR) with their clients. This term identifies an effective relationship that underpins therapeutic processes such as the building of trust and empathy between the therapist and the client [7,8].

However, an effective TR is also associated with the formation of a good cooperation between psychologists and clients defined as *therapeutic alliance* (TA) [7,9]. This has been established as a good predictor of effective psychotherapy [10]. TA consists of 3 critical factors: (1) the sharing of clear expectations and goals by both clients and psychologists; (2) a clear definition of responsibilities, rules, and commitments; and (3) a relationship between psychologists and clients that involves their bonds, mutual trust, and respect [11].

Thus, effective cooperation between psychologists and clients is crucial to the therapeutic processes involved in the therapeutic context and to establish a beneficial TR. A lack of either of these two factors, of TA and TR, prevents psychologists and clients from laying the foundation for clients’ future changes.

The strict correlation between the therapist and client relationship and treatment outcomes has also been recognized in the literature [12-17]. For instance, Lorr [13] conducted an interesting study focusing on the clients’ perception of the TR: he highlighted that the 5 dimensions included in the client-therapist relationship, that is, accepting, understanding, authoritarian, independence-encouraging, and critical-hostile data, are strictly linked to positive or negative outcomes. His research underlines that the relational factors of accepting and understanding are related to clients’ improvements [13]. Another study has implemented the research by Lorr [13], extending the explorations to psychologists’ perceptions. This study found that the therapists’ self-perception of the independence-encouraging dimension was associated with client-rated improvements. Instead, different perceptions and ratings of understanding and accepting factors concerning the TR highlighted negative client and therapist outcomes [18].

Hence, the ability of any medium to support the development of effective TR and TA is therefore critical. Videoconference therapy has been growing, but the COVID-19 pandemic has accelerated the demand for it. There are foreseeable, potential risks in moving therapy to the web by using videoconferencing psychotherapy (VCP). Therapists are not trained to conduct clinical treatments by videoconferencing (VC) as they normally rely on face-to-face interactions; thus, the establishment of a concept termed *presence* becomes essential. This term has been used in VC as a concept that encapsulates 2 elements: (1) the degree to which the web-based experience of another person is analogous to a real-world meeting and (2) the degree to which the user experiences agency and control that impacts the real world [19]. Psychologists are aware of the importance of presence in the development of the therapeutic process and how it is crucial to be connected with clients *on what is going on* [20]. In this paper, we adopted this understanding of presence as a subjective psychological state mediated through VCTs [19]. Consequently, we will provide some pathways to enhance the perception of psychologists’ *presence* in VC sessions. Previous research has indicated that in some cases, therapists have struggled with the absence of physical presence, lack of information from the client’s body language, and reduced visibility of facial microexpressions [21]. Detecting the nuances of the voice is another known problem [22]. Both are vital tools for psychotherapeutic work.

Omodei and McClennan [23] reported that VCP is better received by clients than by psychologists. Although clients assess VCP sessions positively, psychologists perceive the technology as an element that limits the therapeutic processes. Rees and Stone [24] inform that psychologists find VCP inferior to face-to-face consultations in terms of building an effective TA. However, research has revealed that clients’ experiences are more positive than those of psychologists, and little is known about the reasons for psychologists’ major reservations.

In this paper, we focused on the interactions between TA and VC experiences to develop propositions for future research. Through this investigation, we addressed the following question: “How do psychologists and clients experience the TR and TA in videoconference psychotherapy?”

Nonetheless, before going through the VCP studies, we describe a pivotal theoretical framework that has impacted research and practice in psychology. This framework, the General System Theory (GST) [25], will also frame our analysis of VCP. This theory replaced the linear dominant stimulus-response model (robot model); here, the relationship and the interaction were identified in a frame involving an observing subject (the psychologist) and an observed object (the client). The GST reframed this conceiving one broader system of two interacting parts, the psychologist and the client, which influence each other and the whole system. GST helps researchers to detect behavioral patterns, interconnections, and interactions within the TR [26]. By applying this theoretical framework to the VCP context, the broader system mentioned above should also consider the computer as part of the interaction between psychologists and clients. Thus, in this updated framing, we will conduct our analysis considering the computer as an active third party in the communication. Hence, it is crucial to understand how psychologists communicate and deal with the new presence in the VC interaction system, since both might impact the development of therapeutic processes.

In the following sections, we will look at face-to-face psychotherapy, highlighting the impact of presence on the TR. The rest of the paper will propose an overview of the studies related to communication and conducted on computer-mediated communication (CMC). We aim to underline studies relating to cooperative environments where the collaboration and the development of trust among users is crucial for reaching the final goal. Cooperation and trust are essential, key elements for the development of the TA and TR in the clinical context. We will then present the research methodology applied by explaining how the screened, selected articles have been processed. Afterward, we present the literature on both clients’ and psychologists’ experiences of TA and VCP. The last section will be dedicated to the discussion of the results and the presentation of some propositions, followed by the conclusion and suggestions for future research.

### The Influence of Face-to-Face Interaction on the TR

This section introduces face-to-face psychotherapy and the role of presence in developing effective TR and TA. We also expanded on the important role of cognitive and emotional factors in TR.

Research has shown the improvement of clients participating in psychotherapy compared with people who had not received any psychological treatment [27,28]. According to that research, the face-to-face relationship impacts the effectiveness of psychotherapy, enables clients to acknowledge their issues, and creates the basis for a change. In this relationship, therapists act and communicate with their clients by adopting psychotherapy techniques that draw on theories of the TR [29]. Psychologists use all their communication channels when interacting with their clients. One of the most important nonverbal channels is vision, which helps both sides understand each other’s thoughts, intentions, and emotions. Moreover, eye-contact is important to regulate and *control the communication* among people; this aspect of human communication is called meta-communication [30].

As for therapeutic rapport, some studies [11,31,32] suggest that the role of the TR is more significant than either the therapist’s correct identification of the client’s issues or their assessment of the psychological concern. Early studies on the development of effective relationships in face-to-face treatment showed that a few aspects of the TR play a pivotal role, such as “therapist’s personal reactions, [...] the quality of their communications, diagnostic impression and treatment plans” [33]. All these factors, along with the development of TA, should be considered as interrelated rather than independent [34].

Another study noted that psychotherapy is ineffective when therapists struggle to create a relationship based on warm communication and empathy [35]. Thus, TR needs to be formed to determine a change in clients’ lives, and TA must be established to guarantee treatment effectiveness.

**Figure 1:**
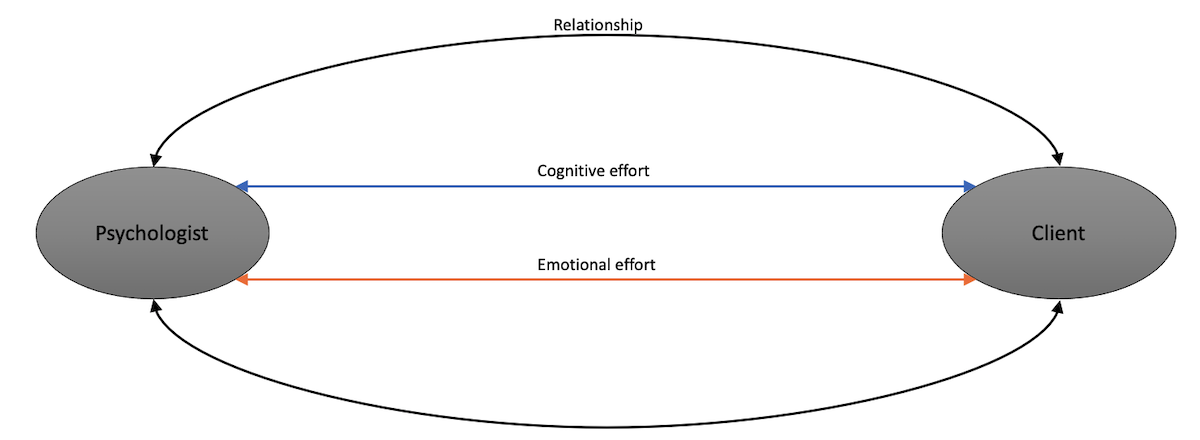
Face-to-face psychotherapy relationship.

In human beings, the interaction processes include 2 aspects: cognition and emotion. According to emerging neuroscience studies, cognition and emotion are 2 separate mental functions that communicate through the mediation of interrelated, separate brain schemes [36]. Tucker et al [37] has shown that cognition and emotion are strictly connected: emotional communication is cognitive work where all the related information is combined and collected from several fonts of the brain. This is important because in every interaction, people process information cognitively and emotionally. Thus, Figure 1 aims to show the circular relationship (not linear but circular, based on the General System Theory [GTS]) linking psychologists and clients during their communication. Although clients are not required to be aware of these underpinning processes, psychologists’ awareness is necessary within the consultation room. Consequently, psychologists process the interaction with their clients taking into account different levels of cognitive and emotional communication but also consider them in an interconnected assessment.

To conclude, the effectiveness of traditional psychotherapy is based on face-to-face interactions that trigger therapeutic and relational processes. An effective sense of presence leads psychologists and clients to interact more successfully: improving communication and the chance to verify the accuracy of the therapists’ diagnosis. By building effective TR and TA, both actors can better achieve the final goal: the client’s wellness. Hence, how do therapists and clients deal with the *absence of presence* in the VCP approach? Does VC technology affect TR and TA formation?

To address these questions, a proper understanding of the concept of trust in CMC is crucial because trust is related to the formation of an effective TR and TA in a therapeutic environment. Hence, analyzing the establishment of trust between people interacting in CMC, it will be possible to gain data regarding the development of trust in computing environments. The presence of trust in face-to-face relationships is necessary for establishing a cooperative rapport aimed toward the achievement of common goals. Trust can then be defined as “a willingness to be vulnerable, based on positive expectations about the actions of others” [38].

In the next section, we present an extended study related to trust in CMC; we aim to gain a proper understanding of trust establishment in the case of relationships mediated by computers.

### CMC Studies in Communication—Trust

Research findings related to trust in CMC are inconsistent. Although studies show that cooperative trust can be easily achieved with a software system [39], other research appears to disagree with that statement, pointing out the difficulty in establishing trust in CMC. According to Handy [40], “trust needs touch,” and other studies highlight the importance of face-to-face interaction clues for building trust. For instance, Nardi and Whittaker [41] claimed that within work environments, traditional face-to-face communication is essential to work efficiently and the CMC affects users in generating *interpersonal bonds*. Drolet and Morris [42] showed how people were more collaborative in face-to-face contact than by phone. Another piece of research [43] revealed that the 6 people involved in the experiment demonstrated higher levels of collaboration when communicating face-to-face rather than mailing.

Some authors [44,45] recognize that the development of trust is very closely related to proximity. Wilson et al [44] explained it as a consequence of sight limitation, which impacts the establishment of trust in remote exchanges: it is harder to detect interaction signals that are potentially useful for achieving the shared task. However, Wilson et al [44] showed how trust is also likely to be developed in CMC, even after a delay: sharing *social information* by CMC involves the commitment of time. They affirm that people are prone to establish social relationships, either through face-to-face communication or through CMC. Wilson et al [44] also explained that trust formation is delayed for groups using computer-mediated systems (CMSs) because the interaction requires almost four times the number of messages transmitted by face-to-face interaction [46,47]. In their experiment, the cognitive and affective trust (in 3 different CMS texts) was negatively affected by the changes in communication media (happening at every meeting) while they increased when passing from CMSs to face-to-face. However, they were lower at the end of the first meeting, but by the end of the third meeting, the levels of trust were similar to face-to-face interaction.

Bos et al [48] analyzed trust development through different communication channels such as face-to-face interaction, VC, 3-way phone conference, and text chat. They confirmed that trust development is possible but unavoidably characterized by a higher delay and fragility compared with face-to-face meetings, even though the respondents subjectively reported high levels of collaboration. Thus, with regard to VC, trust appears to be present but is quite vulnerable. However, it has also been shown that if a spatially faithful VC system (multiview design) is provided, there is no substantial difference in terms of trust between face-to-face and VC group meetings [49].

In summation (Table 1), the literature examined showed that trust establishment is possible but, at the same time, highlights its fragility. However, it should be noted that when we move to psychotherapy, trust cannot be fragile in face-to-face or videoconference [50].

**Table 1:** Trust in computer-mediated communication.

Study	Title	Study design	Study participants	Task	Methodology	Outcome
Bickmore et al [39] (2005)	Establishing the computer–client working alliance in automated health behavior change interventions	3 treatment groups: Control Nonrelational Relational	Healthy participants interested in increasing their physical activity	Interaction with a software	Mixed methods	Trust is reachable even with a software
Bos et al [48]	Effects of four computer-mediated communications channels on trust development	66 groups (3 persons per group) interacting face-to-face and through video, audio, and text chat	People related to the university (mostly students)	Interacting during a social dilemma game: Daytrader	Quantitative	Trust emerges with delay and is fragile
Nguyen and Canny [51]	Multiview: spatially faithful group video conferencing	3 experiments (7 groups of 3 individuals and 1 group of 2 individuals): Partial and full spatial awareness with respect to gaze Same as A with higher attention to gesture Mutual spatial awareness with respect to gaze	Sample from the University of California, Berkeley (undergraduate and graduate students)	The sample was asked to judge the pupils’ direction	Qualitative	Presentation of the multiview design
Nguyen and Canny [49]	Multiview: improving trust in group video conferencing through spatial faithfulness	29 groups of 2 individuals and 37 groups of 3 divided in the following groups: face-to-face groups Directional VC^a^ Nondirectional VC	169 participants (from the Social Science Laboratory, University of California, Berkley)	Modified version of the social dilemma game developed by Bos et al [48]	Mixed methods	Spatial distortion in group meeting by video negatively impacts the development of trust, while trust is established when a spatially faithful VC system is provided
Drolet and Morris [42]	Rapport in conflict resolution: Accounting for how face-to-face contact fosters mutual cooperation in mixed-motive conflicts	2 experiments. Solving conflict in side by side or face-to-face–phone or face-to-face	Experiment 1: 134 master’s students from Stanford University (Department of Business Administration) Experiment 2: 42 persons (master’s and bachelor’s students) from Stanford University	People needed to negotiate having access to nonverbal behavior and cultivating relationships that enable reciprocal collaboration	Quantitative	People were more collaborative in face-to-face interactions rather than by phone

^a^VC: videoconferencing.

Nonetheless, research conducted on CMC provided inconsistent outcomes related to whether the psychotherapy sessions could be face-to-face or CMC. In our previous study [8], we highlighted 2 positions. Based on one view, there are some unknown factors and features of traditional face-to-face communication that cannot be replaced by a computer. For example, according to Russell [52], some functions of communication are strictly correlated to physical presence. Henceforth, physical proximity is deemed necessary [53] by some for certain communication. The second point of view, in contrast, claims that the face-to-face “functions can potentially be choreographed...and potentially analogued”; thus, the computer can replace some signals and be able to reproduce the *functions* typical of face-to-face communication [54].

To conclude, all these studies highlighted the difficulty of establishing a strong trust between users in CMC. Moreover, with regard to the possibility of having psychotherapy sessions face-to-face or through CMC, the literature mentions 2 different points of view; in particular, due to the fragility of trust in CMC, the building of an effective TR is more likely to be compromised.

Before presenting the results of the literature review on the clients and psychologists’ experiences of VCP, we will provide our article selection criteria.

## Methods

### Search Design

This literature review follows the design approach described by Webster and Watson [55] applicable to information system (IS) fields to gather source material for reviewing extant literature. The authors propose identifying the main contributions in leading journals on a selected topic, taking into consideration the interdisciplinary character of the field of IS. Once those contributions to the topics have been identified, Webster and Watson [55] suggest proceeding with a *back and forth* strategy to gather additional studies to be considered in the review. By going *backward*, the authors mean to proceed with a review of the citations included in the articles identified in the first place as leading contributions, to determine which previously published studies should be included in the review process. By going *forward*, the authors mean to use an existing database to identify articles that mention the studies identified in the *backward* phase. This strategy ensures a relatively complete sample of the relevant literature.

### Search Strategy

We started our search on PsycINFO, launching several inquiries. Our strings included keywords selected through the analysis of the works identified in the two prior steps: *VC, psychotherapy, telehealth, online counseling, face-to-face, TR, TA, working alliance (WA), and trust*. Through different combinations of these searches, we obtained thousands of results that were then refined using filters and advanced research tools. We proceeded with a fast screen of the titles to significantly refine the sample of articles, retaining only those that aligned with the scope of our study. This sample was analyzed following previously established eligibility criteria, such as the type of studies (analyses related to the TA and TR experienced by psychologists and clients, and all the studies not covering our topic area), study design (we considered empirical and other research designs including questionnaires, experiments, theoretical papers, etc), data source (leading journals, high class conference proceedings, and the theoretical studies considered significant to our topic), and publication status (we considered studies supported by clear references to journals and places where conference papers have been presented).

**Figure 2:**
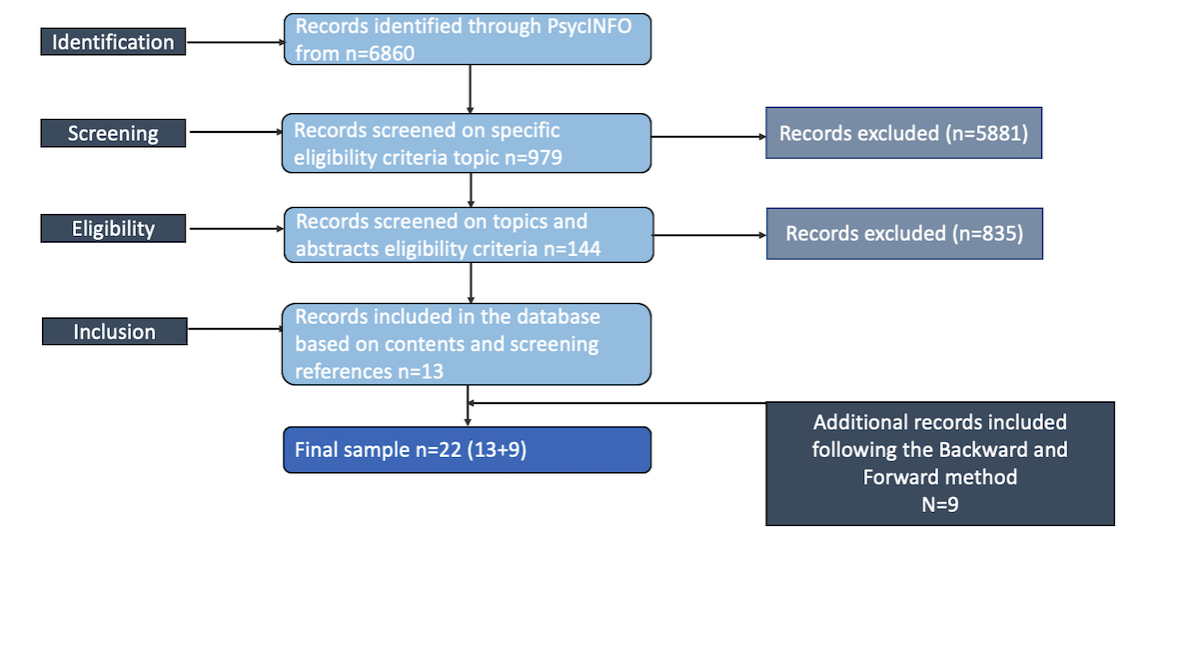
Flowchart. Selection of studies.

Consequently, we started our *backward* and *forward* review process from the analysis of the most relevant papers related to videoconference and psychotherapy and published in leading journals. Simultaneously, we examined the articles’ reference lists to identify prior relevant research and the work of leading authors in the field. By applying the aforementioned criteria, the articles of interest resulted in the final sample of 22 studies (Figure 2). We then proceeded with the inclusion in our analysis of those works that appeared to be fitting the topic of this study. As we were not encountering new concepts and the reading of new papers did not offer any new contribution, we gauged the analysis as concluded. The most relevant studies analyzed are reported in Multimedia Appendix 1 [22,24,56-75].

## Results

### Clients and Psychologists’ Videoconference Experiences

This section focuses on the previous literature reviews that explore the cooperation between clients and psychologists, namely the TA, and processes such as trust and empathy building between both actors of the TR. This section considers the following important studies that have provided significant data, especially considering the needs to deeply comprehend the formation of the TR.

Sucala et al [56] investigated the TR in online web-based therapy; most of their examined research considered TA as a key feature of TR. The results of this study suggest that e-therapy might be comparable with face-to-face interactions, although more studies are necessary to understand the online TR: in fact, its establishment is still doubtful. This study, along with Backhaus et al [57], who investigated potential differences in TR comparing face-to-face and VC modalities, suggested that VCP might be considered a valid alternative to the face-to-face, since clients and psychologists highlighted their general satisfaction. Simpson and Reid [58] explored the TA in VC as a central element for a successful therapy: they claimed there is evidence that suggests that TA is as well supported by VC for both clients and psychologists, although therapists’ rates were often lower than those of clients.

By focusing on these reviews and according to Sucala et al [56], our study aims to explore the possible dynamics hidden in the development of TR by VC. Although research supports VCP as a useful, alternative way to deliver treatments, especially relying on clients’ experiences, there is little information on the ongoing skepticism and difficulties of psychologists. The scarcity of data and the difficulties of researching the establishment of the TR by VC led us to focus on the elements that might change and impact the relationship between psychologists and clients.

By reporting previous studies providing useful insights on clients’ and psychologists’ videoconference experiences, this research will offer a new perspective aimed at understanding the relational dynamics buried in the foundation of the TR by VC. The following investigations suggest positive clients’ experiences, but the literature also reports that psychologists struggle to deliver VC treatments. For this reason, it is essential to understand the different experiences in terms of the psychologist-client relationship.

### Clients’ Videoconference Experiences

Before presenting the following studies, it is important to mention that some research replaced the term TA with WA. The latter, according to Bordin [11], is a *universally applicable* term and refers to people willing to cooperate for a change. Thus, in the context of psychology, the expressions of WA and TA refer to the same meaning and are interchangeable.

In this section, we introduce the previous literature on clients’ experiences of VCP: while there are some doubts about the opportunities to generate interactive VCP features similar to those of face-to-face treatment [59], there is evidence of psychotherapeutic efficiency and a correlation between VCP and healthy behavioral changes [60].

Schopp et al [61] conducted an experiment on 98 adults with cognitive debilities. Half of the sample was treated through face-to-face interactions, while the other half was included in a control group treated through VC. The results showed that clients were generally satisfied, while psychologists rated the face-to-face interactions better than VC. A similar result has been shown in the preliminary study by Storch et al [62], which reported positive results from the young sample affected by obsessive compulsive disorder by VC. However, psychologists expressed their concerns due to the difficulty in establishing the TA, especially with children.

In another study, we noticed that some clients might be influenced by the lack of face-to-face communication, which limits trust establishment. Haberstroh et al [63] focused on web-based counseling sessions, proving the importance of trust for the research sample. Although some participants were encouraged by the lack of face-to-face communication to develop a sense of security as clients, others stated that the same factor had impacted their development of trust. In fact, according to Cook and Doyle [64], in web-based counseling, many obstacles hinder the TA, as nonverbal information is considered vital in generating relationships and intimacy [76] in counseling relationships [77]. The absence of these factors seems to impact the general picture of the relationship and the effectiveness of the treatment.

Nevertheless, with regard to the WA, Day and Schneider [65] analyzed and compared 3 groups treated by face-to-face, telephone, and video communication. The sample concluded at least 5 sessions: while a low level of participation was detected in face-to-face clients, no substantial difference was found in the formation of WA and the session outcomes.

The same result was confirmed by Glueckauf et al [66]. They conducted a study applying VCTs, speakerphone, and face-to-face channels, involving 22 young individuals affected by epilepsy. The research did not attest for any discrepancy in WA inventory and treatment adherence. All participants experienced a significant decrease in the grade of harshness and occurrence.

Studies conducted with participants affected by PTSD showed great results. A study conducted by Germain et al [67] with 46 participants showed that the sample did not present any significant difference in establishing TA. Gray et al [68], in a preliminary study on people living in a rural area, highlighted a positive response from psychologists, crisis center staff, and clients. The same result of feasibility and safety has been noticed by Acierno et al [69] with a large sample of veterans.

Carpenter et al [70] have also shown that TA can be established by VC in a study involving young people (with anxiety) and their families.

From the analyzed studies, although some inconsistencies emerge, it appears that clients could easily establish the TA and a proper relationship with their psychologists, as technology would support them in enhancing interaction, attention, and intimacy.

### Psychologists’ Videoconference Experiences

In this section, we present the psychologists’ VCP experiences, which strongly differ from the clients’ VCP experiences. Indeed, psychologists had concerns about the establishment of a relationship with their clients.

Wray and Rees [71] explored psychologists’ skepticism toward VCT. The authors found that psychologists agree that sessions conducted by VCT are not as effective as the face-to-face ones. They highlight psychologists’ apprehensions about the difficulties to manage clients with personality disorders, suicide instinct, etc, who are harder to manage via video rather than face-to-face, and communication, as they find it difficult to be warm and comprehensive when trying to transmit empathy by VC.

However, while Cohen and Kerr [72] did not find an important difference in the TA when comparing web-based and face-to-face sessions, a study conducted by Hanley [73] registered a different response as psychologists reported dissatisfaction with the TA.

Rees and Stone [24] wanted to further investigate the development of TA in face-to-face versus VCP. In their study, one of the authors replicated with an actor an authentic session, which was video recorded as an face-to-face session and then repeated in a VC session. The session results were identical, and an external psychologist was contacted to prove that both videos of the sessions were satisfactorily comparable. Both videos, 20 min each, were then shown to psychologists. Therapists have been asked to watch both sessions and rate the TA. The overall results highlighted that VC sessions scored lower than face-to-face sessions.

A more recent study [74] captured a range of psychologists’ concerns about the possibility of creating and/or maintaining TA by VC. Another study on parental counseling [75] claimed the overall satisfaction of clinicians and clients. However, some therapists struggled to establish the TR and clients expressed their preference for mixed therapy (face-to-face and VCP). Nonetheless, it was noticeable that psychologists gained confidence throughout the project’s lifetime.

According to Fletcher-Tomenius and Vossler [22], psychologists need to trust their mental picture of clients and vice versa. In their study, therapists pointed out that there are several *elements of uncertainty* referred to in their mental pictures and the only possible solution would be to trust it, although there are no possibilities of understanding whether it is truthful.

Summing up these studies, psychologists’ difficulties in establishing relationships with their clients emerge. These are summarized in Multimedia Appendix 1.

## Discussion

### Principal Findings

In this study, clients and psychologists’ experiences of VCP were presented. The analyzed investigations reported inconsistent results. Some research claims VCP efficiency and its correlation to clients’ beneficial changes [60]. However, despite research having underlined clients’ positive responses in terms of TA and gratification, other research highlights clients’ difficulty in establishing a good TA. The literature on psychologists’ experiences is more consistent. Psychologists express that technology inhibits them from establishing a TR with their clients as effective as in face-to-face therapy.

By examining clients’ VCP experiences, it seems that they take advantage of the VCP in terms of developing TA without experiencing discernible differences with face-to-face psychotherapy [58,65,66] and with higher levels of participation [65]. It seems that digital communication increases intimacy and eases the interaction when compared with a physical context, and this would benefit psychologists and clients in determining the acceleration of the psychotherapy process [78,79]. Moreover, the lack of face-to-face contact seems to encourage clients to develop a strong sense of security [63]. However, some studies in contrast discovered difficulties in establishing a strong TA due to the lack of body language and nonverbal communication [64,76,77].

It appears that the lack of presence might impact the general picture of the relationship and, consequently, the effectiveness of the treatment. Although there are inconsistent data regarding TA establishment, clients are gratified and obtain beneficial results by VCP.

Conversely, considering psychologists’ VCP experience, we observed more consistently negative responses. They feel *limited* by technology, especially with clients with strong mental and emotional disorders. Psychologists feel that it is difficult to be transparently warm, understanding, and empathic [71]. Feedback on the quality of TA in VCP showed a general displeasure among psychologists [24,62,73]. With regard to the experience of VCP, they complain of the lack of information (especially visual data). This leads them to generate and rely on their own *mental picture* of the client’s state due to the *element of uncertainty* and on their internal trust toward the whole process, both about themselves and their clients. They appear to feel unable to adequately *reach* their client via VC [22]. Furthermore, the absence of sight and physical proximity appears to be a key issue in CMC, since it does not allow users to acquire the information needed to establish trustworthy cooperation. As a result, trust appears fragile.

Considering all of the aforementioned studies, the incongruence, and the inconsistency of both clients’ and psychologists’ VCP experiences, we developed a theory aimed at providing a new framework for understanding the relationship connecting both the actors of the VCP interaction. Thus, in the next section, we introduce the model of the invisible third party.

Furthermore, according to the aforementioned studies, we explored the following propositions:

- Proposition 1: VCP might help clients to rapidly reach a strong TA by using digital technologies that enhance communication and intimacy with the psychologist.
- Proposition 2: VCP might require clients to put more emotional than cognitive effort in the relationship with their psychologists.
- Proposition 3: VCP impacts the psychologists’ ability to establish a strong TA because of the lack of information and control over the relationship.
- Proposition 4: VCP might help psychologists establish a strong relationship with their clients by increasing the quantity and quality of information.
- Proposition 5: VCP might impact the confidence of psychologists, as they have to build a mental picture of their clients without proving it.

### Model of the Invisible Third Party

**Figure 3:**
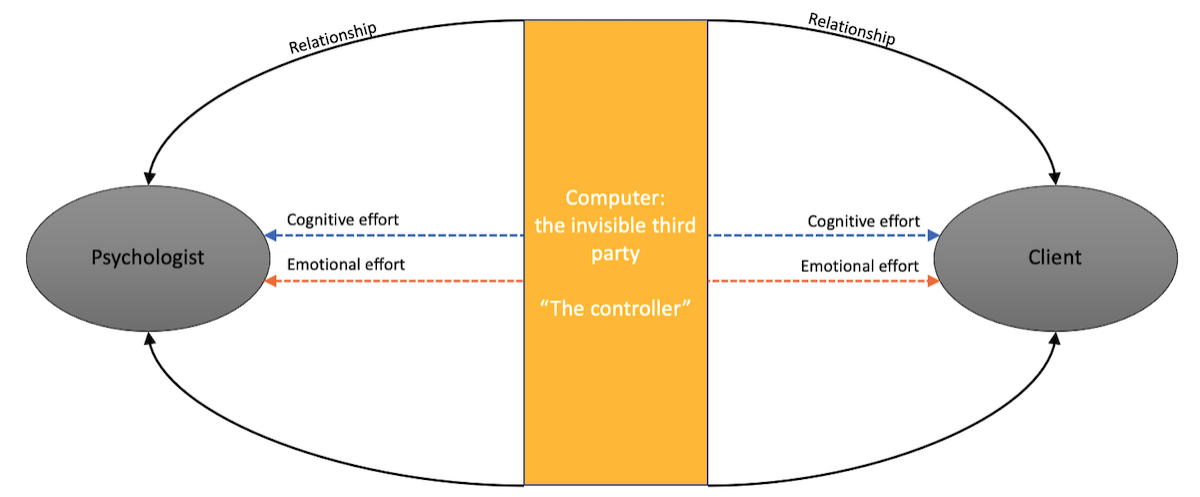
Videoconference psychotherapy relationship model of the invisible third party.

The contribution of this paper draws attention to the role of the computer not only as a vehicle of communication but mostly as a *presence*. Indeed, following the study by Birdwhistell [80], we claim that any person is part of the communication process; we believe that all the components involved in the video communication are part of the system: psychologist, client, and computer. This position combined with the GST [25] leads us to propose a model of the invisible third party (Figure 3). Here, we consider the computer as a new member of the VCP. In this new scenario, the linear stimulus-response model has been replaced by the GST, which includes both psychologists and clients in a broader system of mutual influence. Hence, the computer is to be considered a new member of the circular relationship holding a new influence over the system. However, how does the computer impact the relationship between psychologists and clients?

According to Haley [81], the therapist’s control over the TR is important for successful psychotherapy. Here, control is referred to as an interpersonal dynamic where *each participant is able to control or constrain the behavior of the other*; however, while therapists employ less control than clients according to the relational perspective, they employ extensive *control and influence* in specific and impacting areas with regard to TR [82].

In our model, we introduce the computer as the third invisible party (Figure 3) impacting the dynamics of *control* within the relationship between psychologists and clients. This perspective changes the traditional psychological framing and justifies the lack of confidence of psychologists in working with VC. In VCP there is the technical presence of the computer, which has *technical* control over the relationship between psychologists and clients. In this framework, the computer is not only the interaction vehicle between psychologists and clients, but it is a presence. Thus, the circular interaction of communication and the relationship is impeded and impacted by everything happening outside the psychologists’ control. Therefore, we deduce that the cognitive mental effort might be highly demanding for psychologists who have to work around the new presence and to deal with external technical factors not strictly depending on themselves or their clients. This might require psychologists to create a virtual mental picture of their clients. Moreover, they apparently need to *meta-communicate* bypassing the technology. Meta-communication might require psychologists to make greater cognitive and emotional effort, since they must overcome the obstacles posed by the video.

This new member of the interaction continuously gives new rules to the concept of relationship building. The computer-mediated application of meta-communication might result in high complexity, along with psychologists’ required emotional efforts. Consequently, psychologists might prefer face-to-face psychotherapy to VCP due to the lower cognitive effort required by face-to-face psychotherapy.

According to our model, the following propositions should be explored:

- Proposition 6: VCP might require psychologists a specific cognitive effort, rather than an emotional one, in meta-communication with their clients.
- Proposition 7: VCP might challenge psychologists in dealing with the cognitive overload effort to mentalize and solve the unpredictable presence of the computer, which might control the relationship.

### Conclusions

Studies based on CMC highlighted that communication can be impacted by audio-video quality [83], the lack of body language information [51], and the confusion of users in exchanging gazes, as it is hard to understand where people are looking [84].

Of course, if all these aspects challenge the communication mediated by the technology, they can also have a severe impact on the effectiveness of psychological treatment. Psychologists are trying to provide their support to their clients, even in large organizations [85], through the VCTs, yet the literature on the topic so far has underlined several communication barriers. These obstacles might hinder the establishment of an effective TR.

Our review of the literature on the experiences of clients and psychologists in the VCP highlighted that they have opposite perceptions of TA and TR established by VC. The role of the VCP in supporting psychologists and clients appears particularly crucial, especially in the current pandemic period. The current situation has also required an incremented use of smartphones for VCP, and according to Kim et al [86], there is no substantial difference in using mobile VCP and face-to-face.

Based on the results of this investigation, the model of the invisible third party has been presented; this includes the computer as one of the actors of the psychotherapy relationship. This actor’s role has not been considered so far in studies conducted on VCP. Nonetheless, we argue that its presence in the relationship impacts the development of the elements required for the emergence of empathy, trust, and emotional bonds. These processes are in fact necessary to establish a successful TA and determine VCP positive outcomes. With COVID-19 giving rise to global growth in the adoption of VC platforms, it is more likely that therapists are going to be increasingly relying on VCTs [87]. Initial studies have indicated that therapists, after familiarizing themselves with the VCP and VCTs, might increase their confidence in using such technologies and the quality of their TA and TR [75]. Therefore, it could be argued that therapists need specific training to deal with the features of a computer-mediated interaction with their clients. Based on our investigation in this paper, such training and professional development must include building presence (as defined by Rogers [4-6]) to develop TA and TR through CMC. By using the video as a resource and not as a limitation, psychologists might try to be *present* and connect with their clients *on what is going on.* Finally, future studies should explore if this enforced period of VCP (due to COVID-19) has impacted psychologists’ work, facilitating the development of different therapeutic skills for dealing with their difficulty in *reaching out* to their clients. Furthermore, future research needs to continue to shed light on the role of the computer as an actor in the VC communication between psychologists and clients, leading to the possible development of new technological tools and interfaces to better respond to psychologists’ requirements.

## Appendix

Multimedia Appendix 1 Overview of the studies examined in our analysis.

## Abbreviations

CMC: computer-mediated communication
CMS: computer-mediated system
GST: General System Theory
IS: information system
TA: therapeutic alliance
TR: therapeutic relationship
VC: videoconferencing
VCP: videoconferencing psychotherapy
VCT: videoconference technology
WA: working alliance
