# Supplementary material for: A Perspective on Client-Psychologist Relationships in Videoconferencing Psychotherapy: Literature Review
Source: JMIR Ment Health. 2021 Feb 19;8(2):e19004. doi: 10.2196/19004 (PMC7935652; doi:10.2196/19004)
Supplement: Multimedia Appendix 1 [file mental_v8i2e19004_app1.docx]

| Study | Title | Psychologists | Clients | Study design | Study participant | Task | Methods | Outcome |
| --- | --- | --- | --- | --- | --- | --- | --- | --- |
| Rees and Stone [24] | Therapeutic alliance in face-to-face versus videoconferenced psychotherapy | ✓ | X | - Administering to an independent observer the Penn helping alliance rate scale for both FtF^a^ and VC^b^ groups | 30 Australian Clinical Psychologists | Watching an identical 20 min treatment session FtF or by VC | Quantitative | Psychologists rated a low TA^c^ by VC |
| Fletcher-Tomenius and Vossler [22] | Trust in online therapeutic relationships: The therapist's experience | ✓ | X | - A semistructured interview to investigate the therapist’s experience | 6 online counselors | Going through the therapist’s experience in treating by VC | Qualitative | Trust is a significative element for the TR^d^ and therapeutic web-based treatment |
| Haberstroh et al [62] | The Experience of Online Counselling | X | ✓ | - 5 counseling sessions through designed (with WebCt software) Web sites. | 7 participants counseling students | Supportive web-based counseling | Qualitative | The sample showed problems with technology. Discrepancy among the sample regarding the counseling relationship |
| Day and Schneider [66] | Psychotherapy Using Distance Technology: A Comparison of Face-to-Face, Video, and Audio Treatment | X | ✓ | - Comparing psychotherapy delivered by FtF, video and audio - Several tests and videos have been rated by 3 external payed therapists | 80 clients with numerous matters, from the weight concerns to personality disorders | The therapists worked in 3 different ways: FtF, video and audio. 5 free sessions to real clients | Quantitative | Clients seemed to be more engaged by video and audio rather than FtF |
| Hufford et al [59] | Home-based, interactive videoconferencing for adolescents with epilepsy and their families | X | ✓ | - Study based on 3 different modalities: (1) home-based video-system counseling; (2) home-based speakerphone counseling; (3) videotaped, office-based counseling | 3 adolescents affected by epilepsy and their families | The sessions were focused on detecting data related to comfort, distraction and TA | Quantitative | Positive results in terms of TA and delivery counseling treatment by VC to adolescents who are affected by epilepsy and their families |
| Schopp et al [60] | Telehealth and neuropsychological assessment: New opportunities for psychologists | ✓ | ✓ | - The sample was treated FtF and by VC | 98 adult people having cognitive debilities | Understanding psychologists’ prospects | Quantitative | Clients were generally satisfied while psychologists rated a better score for FtF rather than for video. Telehealth was cheaper than FtF. Problems related to ethical issues, such as billing and licensure |
| Cook and Doyle [63] | Working alliance in online therapy as compared to face-to-face therapy: Preliminary results | X | ✓ | - The sample was treated in online therapy (email, chat, and audioconferencing) as compared with a representative FtF sample | 14 females and 1 male and 5 therapists (1 male and 4 females) | Measuring WA^e^ | Quantitative | The small sample rated the WA higher online rather FtF |
| Simpson and Reid [57] | Therapeutic alliance in videoconferencing psychotherapy: A review | ✓ | ✓ | - Systematic review on TA in VCP^f^ | X | Measuring TA | Qualitative | Clients rated high levels of TA while psychologists rated TA levels not as high as their clients |
| Cohen and Kerr [73] | Computer-mediated counselling: An empirical study of a new mental health treatment | ✓ | ✓ | - Clients received counseling sessions either FtF or computer-mediated | 24 undergraduate students and 6 graduate counselors | Measuring anxiety and positions regarding counseling | Quantitative | Clients demonstrated to alleviate their anxiety levels |
| Hanley [74] | The Therapeutic Alliance in online youth counselling. | X | ✓ | - This study is focused on Kooth^g^ | X | Evaluating the TA in computer-mediated sessions between adolescents and therapists | Mixed methods | 79% of the adolescents said that the alliance was of a medium or high-level quality. Moreover, the interviews stressed the importance of finding a suitable match with a therapist to build a strong alliance |
| Bambling et al [58] | Online counselling: The experience of counsellors providing synchronous single‐session counselling to young people | ✓ | X | - Counselor answers were organized into privacy and an emotionally safe environment; communication through text; and time | 26 counselors | Observation of counselors delivering online treatments to young people; assessment of the communication environment; and the counselors’ ability to deal with communication problems | Qualitative | Counselors informed the main advantage was the reduction of emotional distance to the therapist, while the main weaknesses were the reduced nonverbal information and the time in finalizing the intervention |
| Glueckauf et al [67] | Telehealth: The new frontier in rehabilitation and health care | X | ✓ | - Counseling through video, audio, or FtF | 39 youths with epilepsy (with parents) | Assessing WA, problem severity and frequency scale, social skills rating system scale and treatment adherence | Quantitative | Videophone and speakerphone treatments are efficient for dealing with family problems as in-person counseling. There has been a general improvement with youths but not substantial behavior change |
| Wray and Rees [72] | Is there a role for videoconferencing in cognitive–behavioural therapy? | ✓ | X | - Therapists were casually assigned to watch an undistinguishable FtF or VC psychotherapy session | 30 psychologists | Assessing psychologists’ positions, which might influence their availability to use technology and their opinion concerning the technology effects on the TA | Qualitative | Mental health providers pointed out that VCP would be less efficient than FtF therapy. Moreover, psychologists were concerned about the TA and the clients’ perception of the therapist *as warm, empathic, sensitive, and understanding* |
| McClellan et al [75] | Clinician Telehealth Attitudes in a Rural Community Mental Health Center Setting | ✓ | X | - Administering a survey | 100 clinicians (psychologists, clinical social workers, therapists, or other mental health experts) | Test the attitude of clinicians to work remotely | Mixed methods | Clinicians are worried about establishing and preserving TA |
| Germain et al [68] | Assessment of the Therapeutic Alliance in Face-to-Face or Videoconference Treatment for Posttraumatic Stress Disorder | X | ✓ | - Psychotherapy delivered by VC or FtF | 46 clients affected by PTSD^h^ | Evaluate the TA | Quantitative | The VC and FtF sample of participants did not show significant difference in terms of TA |
| Carpenter et al [71] | Working from home: An initial pilot examination of videoconferencing-based cognitive behavioural therapy for anxious youth delivered to the home setting | X | ✓ | - Home-delivered psychotherapy | 11 young people (and their families) affected by anxiety | To test the feasibility and families’ acceptance | Quantitative | The result of this study showed its viability |
| Gray et al [69] | Provision of evidence-based therapies to rural survivors of domestic violence and sexual assault via telehealth: Treatment outcomes and clinical training benefits | ✓ | ✓ | - VCP to people with PTSD | 21 participants (all female) | Evaluate benefits on therapist, center staff and clients’ satisfaction receiving VCP | Quantitative | Positive results in matching the needs of rural clients and general satisfaction from psychologists and center staff |
| Acierno et al [70] | Behavioural activation and therapeutic exposure for posttraumatic stress disorder: A noninferiority trial of treatment delivered in person versus home‐based telehealth | X | ✓ | - Home-based vs in-person psychotherapy to veteran people affected by PTSD | 265 participants | Assess behavioral activation and therapeutic exposure, an evidence-based psychotherapy for PTSD and Major Depression (MD) | Quantitative | Results suggest a certain feasibility and safety in delivering psychotherapy to veterans affected by PTSD and depression. Results are in line with FtF sessions |
| Storch et al [61] | Preliminary Investigation of Web-Camera Delivered Cognitive-Behavioural Therapy for Youth with Obsessive-Compulsive Disorder (OCD) | ✓ | ✓ | - VCP with young people affected by OCD^i^ | 31 participants | Evaluate the response from the young sample affected by OCD | Quantitative | VCP showed positive results in this preliminary study in reducing symptoms of OCD in children and adolescents |
| Owen [76] | Feasibility and acceptability of using telehealth for early intervention parent counselling | ✓ | ✓ | - VCP for parent counseling | 9 parents, 4 psychologists, and 9 social workers | Evaluate the practicability and appropriateness of the VCP with parents | Qualitative | Clinicians have shown an increase in confidence in using VC and parents experience a general satisfaction in self-efficacy, promoting the mixed therapy session: FtF and VCP |
| Sucala et al. [55] | The Therapeutic Relationship in E-Therapy for Mental Health: A Systematic Review | ✓ | ✓ | - Systematic review on the TR | X | Assess the TR in e-therapy | X | e-therapy appears similar to FtF psychotherapy |
| Backhaus et al [56] | Videoconferencing Psychotherapy: A Systematic Review | ✓ | ✓ | - Systematic review on the VCP | X | Assess the feasibility of VCP | X | General satisfaction from users and outcomes similar to FtF therapy |
